# An improved graph factorization machine based on solving unbalanced game perception

**DOI:** 10.3389/fnbot.2024.1481297

**Published:** 2024-12-04

**Authors:** Xiaoxia Xie, Yuan Jia, Tiande Ma

**Affiliations:** ^1^BYD Company Limited, Shenzhen, China; ^2^School of Statistics, Renmin University of China, Beijing, China; ^3^School of Computer Science and Technology, Xinjiang University, Urumqi, China

**Keywords:** machine learning, mobile game user evaluation, quality of experience, factorization machine, graph neural network

## Abstract

The user perception of mobile game is crucial for improving user experience and thus enhancing game profitability. The sparse data captured in the game can lead to sporadic performance of the model. This paper proposes a new method, the balanced graph factorization machine (BGFM), based on existing algorithms, considering the data imbalance and important high-dimensional features. The data categories are first balanced by Borderline-SMOTE oversampling, and then features are represented naturally in a graph-structured way. The highlight is that the BGFM contains interaction mechanisms for aggregating beneficial features. The results are represented as edges in the graph. Next, BGFM combines factorization machine (FM) and graph neural network strategies to concatenate any sequential feature interactions of features in the graph with an attention mechanism that assigns inter-feature weights. Experiments were conducted on the collected game perception dataset. The performance of proposed BGFM was compared with eight state-of-the-art models, significantly surpassing all of them by AUC, precision, recall, and F-measure indices.

## 1 Introduction

Mobile games gained a large share of global business, especially during the COVID-induced dead season for other entertainment businesses and activities. Game-related services, from run to finish, interact with each other in multiple directions. The complex functionality of the game user during play requires multiple services to reach together, which involve different functions. The application runs by invoking the most appropriate ones from many alternative services to be combined. In a real Internet environment, multiple service providers usually offer services with the required functionality. These services are distributed differently and hosted on servers in different user regions. These many services are combined through network selection and application invocation to realize the complex functionality the user requires. Therefore, how the customer can judge the most suitable quality service is the key to improving the gaming user’s perceived experience.

When investigating how service quality affects user experience, it is necessary to consider the influence of user and environmental factors such as the user’s level of play, game mechanics, game team, and individual performance. The main idea of existing studies on game user perception is to clarify multiple dimensions of interrelated game perceptions, then establish an objective and easy-to-measure correlation mapping between service quality indices and experience quality, fully consider the influence of other dimensions on the correlation, and finally assess or predict game perceptions using the objective features, to achieve the purpose of optimizing game perceptions. However, these studies need to pay more attention to the importance of features other than service quality features on the outcome of game perception.

This study aims to mitigate some deficiencies of existing algorithms by an alternative approach. To this end, a new method called Balanced Graph Factorization Machine (BGFM), which considers the data imbalance and the importance of high-dimensional features, is elaborated and tested. The data categories are first balanced by Borderline-SMOTE oversampling, and then features are represented naturally in a graph-structured way. The highlight is that the BGFM contains interaction mechanisms for aggregating beneficial features. The results are represented as edges in the graph. Next, BGFM combines factorization machine (FM) and graph neural network strategies to concatenate any sequential feature interactions of features in the graph with an attention mechanism that assigns inter-feature weights. The main highlights in this paper are listed as follows:

The strengths and weaknesses of FM and GNN in modeling feature interactions are analyzed. To solve their problems and take advantage of their strengths, a new model for feature interaction modeling, BGFM, is proposed, which bridges the gap between GNN and FM. The features in the graph are the nodes, and the two-by-two interactions between features are the edges connecting the nodes, making it possible to solve the FM problem by taking advantage of the strengths of GNN.

The similarity between the computed feature interactions of the attention mechanism is introduced to ensure the robustness of LTFM. This enhances the positive effects of effective features while reducing the negative effects due to biased features.

We conducted several experiments on the QoE dataset. The results show that the proposed BGFM performs well and outperforms the existing methods.

## 2 Related work

Previous studies of game user perception have focused only on the correspondence between QoS parameters and game QoE (Wattimena et al., 2006; Koo et al., 2007; Denieffe et al., 2007). This was initially done using linear models (e.g., logistic regression and generalized regression) to generate user game perception scores (Pornpongtechavanich et al., 2022). User-perceived assessment models based on machine learning techniques, such as QoE modeling using SVM to construct prediction models (Suznjevic et al., 2019), have become a research hotspot as they effectively predict user perception. These models ignore useful but unseen feature interactions in the data, as evidenced by the effectiveness of hidden variable models (Sun et al., 2013). Factor decomposition machines (Rendle, 2010) provide a general-purpose predictor to efficiently model higher-order interactions between interpreted features within linear time complexity.

Yang et al. (2021) transformed location information into neighborhood information and added it into a factor decomposition machine to propose the LBFM model. More recently, Wang et al. (2022) proposed an LDFM model using information entropy and location projection of users and services. While the above algorithms extend the dataset somewhat, different cross-cutting features are not distinguished, making the model performance fluctuate. He and Chua (2017) proposed neural factorization machines (NFM) for sparse predictive analytics. Xiao and Ye (2017) thus introduced the neural network strategy on top of the previous ones and proposed the AFM model, which distinguishes between different second-order feature combinations through the attention mechanism. Hong et al. (2019) proposed interaction-aware factorization machines for recommender systems, considering that perceived data sparsity can lead to fluctuations in model performance. To the best of the authors’ knowledge, the only data-driven study of game user perception that considered the effects of multiple factors has been reported in our previous paper (Xie and Jia, 2022), which introduced the location-time-aware factorization machine based on fuzzy set theory for game perception (LTFM).

Despite some progress in the relevant research, two major aspects of the problem need further clarification. On the one hand, poor game user perceptions are a minority occurrence, similar to positive samples required for trade fraud risk prediction in banks. This inevitably runs into the problem of data imbalance. The collected game user perception data are categorized into three evaluation categories: excellent, good, and poor, with an approximate ratio of 5:1:1. In LTFM, the data are divided by tiers to reduce the impact of data imbalance on the overall performance of the algorithmic model. Although the model outperformed others, there is much room for its improvement in several aspects.

On the other hand, a factorial decomposition machine is a model for modeling interaction features. The core of FM is to learn the uniquely hot-coded features corresponding to the hidden vectors, and then the interaction between features is modeled by the inner product of vectors (Rendle, 2010). FM has been used in Cheng et al. (2016) and Guo et al. (2017), exhibiting at least two weak points: (i) it failed to capture higher-order feature interactions, and (ii) it assigned the same weights to all feature interactions, overfitting the model by useless interactions (Zhang et al., 2016; Su et al., 2021). Attempts have been made to transform FM to learn higher-order feature interactions by introducing deep neural networks (DNNs). Neural Factorization Machine (NFM) combines DNNs and dual interaction layers to obtain information about higher-order feature interactions (He and Chua, 2017). Wide & Deep learning model, and DeepFM model combine shallow and deep structures to achieve multi-order feature interactions (Cheng et al., 2016; Guo et al., 2017). However, implicit learning models introduced into DNNs are usually weakly interpretable, while Graph Neural Networks (GNNs) provide a lucrative alternative for grasping higher-order interactions between features (Zhang C. et al., 2021; Hamilton et al., 2017). The core technical point of GNN is to achieve a higher learning rate by accumulating layer by layer and aggregating multidimensional relevant features. As a result, higher-order interactions between features can be explicitly encoded into the embedding, which inspired this study.

All in all, there is a great need to evaluate the perceived experience of game users. There are two main advantages of the proposed BGFM over previous studies:

Treating features as nodes and two-by-two interactions between features as edges mitigates the problem of comprehensively combining GNN and FM, making it possible to solve FM problems via GNN.

The attention mechanism assigns different weights to different features interactively to enhance the utilization of effective features and reduce the probability of deviant features.

## 3 Proposed method

To address the above algorithmic pain points in game perception research, we propose the Balanced Graph Factorization Machine (BGFM) model. To this end, the overall framework of BGFM is decomposed, and the overall working principle of BGFM is summarized. The BGFM firstly chooses Borderline-SMOTE to solve the problem of unbalanced distribution of training data, which leads to fluctuation of model performance. Then, we focus on how to model higher-order beneficial feature interactions. For this purpose, we design a special mechanism in BGFM, which can be split into two main parts: the selection of beneficial interaction features and interaction aggregation. The implementation principles of these two parts are described in detail. Finally, the model-based predictions and the model optimization are discussed.

### 3.1 BGFM

**Figure 1:**
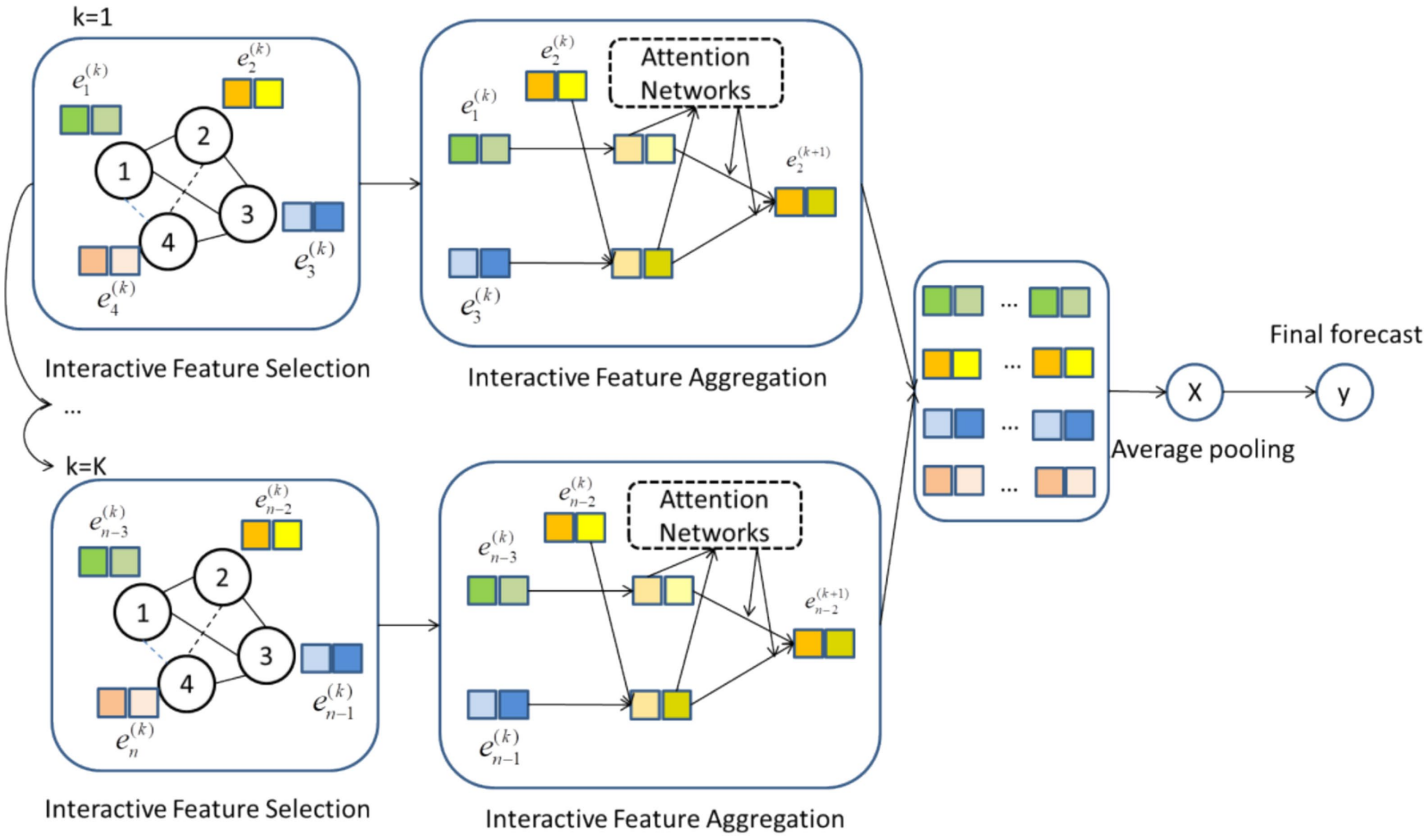
Network structure of BGFM.

Figure 1 shows the network structure of BGFM. The graph flexibly represents higher-order associations between features. Edges in BGFM are useful feature interactions obtained by model aggregation. After resolving the data imbalance, beneficial feature interactions are selected. After learning by the attention mechanism, different feature interactions are given different weights and jointly output for final prediction.

The BGFM will update the network step by step. The input values are processed through feature embedding as the initial data for BGFM, as $e_{i}^{\left(1\right)}=e_{i}$ , where $e_{i}^{k}$ is the latest feature embedding for the k -th layer. The model initially has no pre-input edge information, so edges are first obtained by the interaction selection component. The resulting edge information is then aggregated to update the feature embeddings in the remaining regions.

Existing methods for unbalanced data learning can be divided into three categories. BGFM uses a data-level solution, while others need more flexibility and robustness. BGFM greatly simplifies the workload of model training, improves efficiency, and gets various classifiers. Borderline-SMOTE is an algorithm extended for SMOTE. It considers the effect of noisy samples, and the algorithm uses only a few classes of samples with the attribute Danger on the border to obtain new samples, yielding a balanced distribution of the training sample set. After solving the problem of perceived data imbalance, two main components exist in each layer of BGFM. Both of them are described in detail next.

### 3.2 Interaction feature selection

We devised a mechanism to obtain favorable pairwise feature interactions in the paper. The mechanism is the inference of connections between perceptual features through the graph structure, which models higher-order connections between features. However, the edges connecting two nodes $\left(v_{i},v_{j}\right)\in E$ exist deterministically, greatly simplifying the selection process compared to the direct introduction of gradient descent-based optimization techniques.

This limitation is resolved by replacing the set of edges E by heightened neighbors P , which $P_{ij}$ is explained as the likelihoods of $\left(v_{i},v_{j}\right)\in E$ . It shows that the interaction between the features is very important. A different graph structure $P^{\left(k\right)}$ needs to be learnt at each k -th layer and comparing it with the previously derived graph. These treatments provide higher performance. Specifically, each layer of the model’s graph structure is fixed, culminating in fixed-form outputs. However, our model is characterized by adaptive learning and can model associations of beneficial features.

This section aims to design a metric function to obtain beneficial feature interactions. The $P_{ij}\left(v_{i},v_{j}\right)$ metric function calculate $f_{s}\left(e_{i},e_{j}\right)$ s the weights of the edges. NMF-based functions are used to evaluate the edge weights (He and Chua, 2017). The product of elements of these feature vectors is converted into a scalar using Multilayer Perception (MLP) with one hidden layer, which can be calculated as Equation 1.


(1) $$f_{s}\left(e_{i},e_{j}\right)=\sigma \left(W_{2}^{s}\delta \left(W_{1}^{s}\left(e_{i}⊙e_{j}\right)+b_{1}^{s}\right)+b_{2}^{s}\right)$$

where $W_{1}^{s}$ , $W_{2}^{s}$ , $b_{1}^{s}$ and $b_{2}^{s}$ are the inputs to the multilayer player. ReLU and Sigmoid activation functions are represented by $\delta \left(\cdot \right)$ and $\sigma \left(\cdot \right)$ , respectively. It is worth noting the order of the inputs to $f_{s}$ is invariant, as $f_{s}\left(e_{i},e_{j}\right)=f_{s}\left(e_{j},e_{i}\right)$ . The same pair of nodes have the same edge weights at this point. This successive graph structure modeling allows the gradient to backpropagate. Since there is no truth graph structure, the gradient here is defined by the deviation between the model’s estimated and actual values. Feature interactions are treated as one, and the weights are estimated using MLP. Euclidean distance or other distance metrics can also be chosen (Zhang W. et al., 2021).

### 3.3 Interaction aggregation

After selecting the beneficial feature interactions, the feature representation is updated by performing an interaction aggregation operation. For the target feature node $v_{i}$ , the attention coefficient of each feature interaction is measured while aggregating its beneficial interactions with its neighbors. The learnable projection *a* and nonlinear activation function LeakyReLU are applied to measure the attention coefficients as Equation 2.


(2) $$c_{ij}=\mathrm{LeakyReLu}\left(a^{T}\left(e_{i}⊙e_{j}\right)\right)$$

This implies the significance of interactions between features $v_{i}$ and $v_{j}$ In this paper, we only compute $c_{ij}$ of the node $j\in N_{i}$ . $N_{i}$ represents the neighbors of the node $v_{i}$ , which is the sum for features that are useful to interact with $v_{i}$ . In the paper, the following function Softmax is used to normalize them in all choices of j , as shown in Equation 3.


(3) $$a_{ij}=\mathrm{Softmax}\left(Y_{ij}\right)=\frac{e^{Y_{ij}}}{\sum_{k=1}^{K}e^{Y_{k}}}$$

where $Y_{i}$ is the output value of the i -th node, the output values of the multi-classification range vary from 0 to 1; k is the number of nodes that the network finally outputs, that is, the number of categories that can be classified. This makes it easy to compare the coefficients obtained between different feature nodes. After obtaining the normalized attention coefficients, the linear and nonlinear combinations of links between features are computed as subsequent new feature inputs as Equation 4.


(4) $$e_{i}^{1}=\sigma \left(\underset{j\in N_{i}}{\sum }a_{ij}b_{ij}W_{a}\left(e_{i}⊙e_{j}\right)\right)$$

where $a_{ij}$ measures the attentional coefficient of feature i and feature j interactions, and $b_{ij}$ indicates the probability that such feature association is helpful. $a_{ij}$ The attention coefficient is computed via the soft-attention mechanism and $b_{ij}$ is computed by the hard-attention mechanism. The information about the selected feature interactions is controlled by multiplying them and making the input values of the feature interaction selection mechanism learnable by gradient backpropagation.

To capture the diverse polysemy of feature interactions in different semantic subspaces and to stabilize the learning process, this paper extends our mechanism by applying multi-head attention (Li et al., 2017; Marcheggiani and Titov, 2017; Wang et al., 2019). Specifically, *H* individual attention mechanisms perform the update of Equation 4 and then concatenate these features to produce an output feature representation as Equation 5.


(5) $$e_{i}^{2}=\overset{h}{\underset{H}{∥}}\sigma \left(\underset{j\in N_{i}}{\sum }a_{ij}b_{ij}W_{a}^{h}\left(e_{i}⊙e_{j}\right)\right)$$

where || denotes the cascade, $a_{ij}$ is the normalized value obtained through the h -th attention machine with $W_{a}^{h}$ is the linear transformation matrix of the former. Optionally, the feature representation can be updated using average pooling, as shown in Equation 6.


(6) $$e_{i}^{3}=\sigma \left(\frac{1}{H}\overset{H}{\underset{h=1}{\sum }}\underset{j\in N_{i}}{\sum }a_{ij}b_{ij}W_{a}^{h}\left(e_{i}⊙e_{j}\right)\right)$$

### 3.4 Forecasting and improvement

The results for k -th layer is $\left\{e_{1}^{\left(k\right)},e_{2}^{\left(k\right)},\ldots ,e_{n}^{\left(k\right)}\right\}$ , which is a collection of n feature representation vectors. Because the representations acquired in multiple layers model different orders of interactions, they play different roles in the ultimate result. Thus, they are connected in series to get the definitive expression for every feature (Beck et al., 2018) as Equation 7.


(7) $$e_{i}=e_{i}^{1}∥\cdots ∥e_{i}^{n}$$

Finally, all the feature vectors are pooled equally to get the result at the graph level, and the final prediction is made using the projection vector p . The obtained results are computed using Equations 8, 9:


(8) $$e=\frac{1}{n}\overset{n}{\underset{i=1}{\sum }}e_{i}$$

(9) $$\hat{y}=p^{T}e$$

## 4 Results and discussion

### 4.1 Research data

The research focuses on exploring the effects of multiple influencing factors (including user, system, and contextual ones) on the perceived QoE of game users. A general taxonomy of the various factors in the literature is drawn upon, and further references are made to the taxonomy of existing game-related studies in terms of game QoE. Finally, an empirical test method is derived (Pornpongtechavanich et al., 2022; Jiang et al., 2019). Specifically, this gaming dataset considers the effects of three different system factors (latency, packet loss rate, jitter, and additional network parameters), user skills (user-personal factors in terms of gaming experience), and context (in terms of action categories and social context). The game entity under study is Glory of Kings, a game in which the interaction is mainly based on the UDP protocol, which requires a high level of real-time and user engagement. Due to the lack of a dataset of user game perception, the testing process in the study’s laboratory environment was determined after reviewing the relevant literature.

In joint efforts of team members and participants, data from 789 games were collected, with each piece of data representing three dimensions of user, service, and environmental data. Each dataset has 21 features, consisting of 4 pieces of user data (player ID, age, gender, and skill level), 16 pieces of in-game and post-game service data, and a user-perceived score for the last one.

### 4.2 Comparison algorithms

In this paper, to demonstrate the effectiveness of the proposed algorithm, we compared it with algorithms from four categories: (A) linear methods, (B) FM-based methods, (C) DNN-based methods, and (D) aggregation-based methods. The specific eight comparison algorithms include LR (A), Standard FM (Rendle, 2010) (B), NFM (He and Chua, 2017) (C), AFM (Xiao and Ye, 2017) (B), AutoInt (Song et al., 2020) (D), Fi-GNN (Cui et al., 2019) (D), InterHat (Li et al., 2020) (D).

LR is a linear regression, modelled using only a single feature; Standard FM is able to model second-order interaction links of features; NFM designed a dual interaction layer and DNN to handle nonlinear features and model higher order feature interactions; AFM introduces the attention mechanism to give weight to the interaction of different features; AutoInt is to improve the efficiency of the model in learning higher-order feature interactions through self-attentive networks; Fi-GNN uses gated graph neural networks to model higher-order feature connections as fully connected graphs; InterHat uses the attention machine to select features, and raw feature multiplication produces higher-order feature interactions; LTFM is an extended FM-based model that considers the effects of temporal and spatial information projections and feature interactions on the final game perception results.

### 4.3 Evaluation of performance indices

The following five assessment metrics are used in the experiments of game user perception evaluation: AUC (Gospodinova et al., 2023; Li et al., 2022), Precision (Annadurai et al., 2024; Chan et al., 2024), Recall (Wang et al., 2024; Zheng et al., 2024; Hong et al., 2024), and F-measure (Li et al., 2024a; Li et al., 2021; Li et al., 2023a; Guo et al., 2024a; Guo et al., 2024b; Ma and Tong, 2024; Sultan et al., 2024; Li et al., 2024b; Li et al., 2024c; Li et al., 2023b; Li et al., 2023c).

The AUC curve is taken as the area under the ROC curve. The larger the value, the better the model performance. Precision is used to calculate the proportion of correct predictions among all samples with positive predictions. Recall is the ratio of positive class samples correctly judged by the classifier to the total number of positive class samples. Usually, accuracy is inversely proportional to recall. A composite metric, F-measure, is introduced to balance the effects of precision and recall and to evaluate a classifier more fully. When both precision and recall are high, the value of F-measure is high.

### 4.4 Results and analysis

The performance of the BGFM model after balancing the data was first analyzed experimentally, as shown in Table 1. It is concluded that there is an improvement in the BGFM model performance compared to the standard FM.

**Table 1:** Quantitative performance comparison of standard FM and balanced model.

Model	AUC	Precision	Recall	F-measure
Standard FM	0.7549	0.7189	0.7215	0.7008
BGFM (balanced data)	**0.7760**	**0.7575**	**0.7402**	**0.7334**

The bold values are meant to be the best performing performance indicators in the table.

It can be deduced from Table 1 that FM performs well for sparse feature data. However, since poor game perception is a minority class occurrence, FM is impairing the correctness of the final judgment by judging the minority class as the majority class. Due to the data volume limitation, we consider preprocessing the data and de-rationalizing the generation of new data from the existing data to achieve the result that the data classes to be judged are basically the same. The balanced data is then imported into our model. The results show that using Borderline-SMOTE oversampling to balance the data category distribution is beneficial for the final perceptual evaluation.

The performance comparison of these methods on the game-aware dataset is shown in Table 2, from which the following observations are obtained: the BGFM proposed in this chapter achieves the best performance on the game perception dataset. The enhanced efficiency of the BGFM compared to the four classes (A, B, C, and D) of methods is particularly significant. BGFM employs a mechanism for choosing and aggregating beneficial feature interactions, and its performance is superior and easy to manage. Taking all aspects together, BGFM is superior to existing algorithms. In the following, the four types of algorithms, A, B, C, and D, will be specifically analyzed.

**Table 2:** Quantitative comparison of different algorithms.

Model	AUC	Precision	Recall	F-measure
LR	0.7288	0.7960	0.7975	0.7802
Standard FM	0.7549	0.7189	0.7215	0.7008
NFM	0.7721	0.7789	0.7595	0.7778
AFM	0.7862	0.8052	0.7848	0.7972
AutoInt	0.7908	0.8158	0.7975	0.8012
Fi-GNN	0.8014	0.8207	0.7975	0.8010
InterHat	0.8017	0.8259	0.8101	0.8069
LTFM	0.8093	0.8328	0.8228	0.8178
BGFM	**0.8305**	**0.8760**	**0.8360**	**0.8453**

The bold values are meant to be the best performing performance indicators in the table.

Aggregation-based methods outperform the other three classes of models, demonstrating the advantages of selection strategies in getting higher-order relationships. However, the LTFM model performance still performs well, suggesting that the importance of projected information that considers both temporal and spatial information interacting with and capturing features for the final game perception results is favorable for the final perceptual evaluation. However, this model only expands the data dimensions and captures hidden feature interactions based on the properties of FM for sparse data, which fails to address the performance fluctuations caused by the imbalance of data categories and captures higher-order beneficial feature interactions. The BGFM solves these problems well.

Compared to the powerful aggregation-based baseline AutoInt and Fi-GNN, BGFM still offers a significant performance improvement and can be considered important for game-aware prediction tasks. This enhancement is due to the combination of GNN with FM. Treating features as nodes, two-by-two interactions between features as edges, and each input as a graph, GNN’s aggregation strategy solves two of FM’s problems: suboptimal feature interactions that lead to model overfitting and the difficulty of modeling higher-order feature interactions. GNN introduces the concept of feature interaction and a beneficial interaction selection method that greatly improves the model’s performance.

The attention mechanism assigns different weights to interactions. AFM outperforms FM, demonstrating the necessity of considering feature interaction weights. Although NFM uses DNNs to model higher-order interactions, they do not ensure an improvement over the base model and the improved model with the addition of an attention mechanism, possibly because of their implicit feature interaction learning approach. AutoInt performs better than AFM because the multi-head attention mechanism in the model takes into account the richness of feature interactions in multiple spaces.

## 5 Conclusion

This study bridges FM and GNN approaches, yielding a new BGFM model. It exploits the respective strengths of FM and GNN, attempting to compensate for their individual deficiencies. Beneficial feature interactions are selected at each layer of BGFM and considered edges in the graph. The interactions are then encoded as feature representations using the neighborhood interaction aggregation operation. The model adds higher-order feature learning at each layer, and the layer depth determines the median result. This leads to the conclusion that our model can learn the highest-order feature interactions. The BGFM learns higher-order interactions between features and provides high interpretability of model results. The experimental results prove that the proposed BGFM outperforms eight state-of-the-art models to a large extent.

## Data Availability

The raw data supporting the conclusions of this article will be made available by the authors, without undue reservation.
